# Developing VISO: Vaccine Information Statement Ontology for patient education

**DOI:** 10.1186/s13326-015-0016-2

**Published:** 2015-05-01

**Authors:** Muhammad Amith, Yang Gong, Rachel Cunningham, Julie Boom, Cui Tao

**Affiliations:** School of Biomedical Informatics, University of Texas Health Science Center, 7000 Fannin St, Houston, 77030 TX USA; Immunization Project, Texas Children’s Hospital, 1102 Bates, Houston, 77030 TX USA

**Keywords:** Biomedical informatics, Vaccines, Vaccine Information Statements, Knowledge based systems, Ontology, Ontology construction

## Abstract

**Objective:**

To construct a comprehensive vaccine information ontology that can support personal health information applications using patient-consumer lexicon, and lead to outcomes that can improve patient education.

**Methods:**

The authors composed the Vaccine Information Statement Ontology (VISO) using the web ontology language (OWL). We started with 6 Vaccine Information Statement (VIS) documents collected from the Centers for Disease Control and Prevention (CDC) website. Important and relevant selections from the documents were recorded, and knowledge triples were derived. Based on the collection of knowledge triples, the meta-level formalization of the vaccine information domain was developed. Relevant instances and their relationships were created to represent vaccine domain knowledge

**Results:**

The initial iteration of the VISO was realized, based on the 6 Vaccine Information Statements and coded into OWL2 with Protégé. The ontology consisted of 132 concepts (classes and subclasses) with 33 types of relationships between the concepts. The total number of instances from classes totaled at 460, along with 429 knowledge triples in total. Semiotic-based metric scoring was applied to evaluate quality of the ontology.

## Introduction

In the present information age, patients are afforded many options to educate themselves on vaccines. Some of these options included valid and reputable websites, books, and other media sources while other options may appear reputable but are not. Regardless of the source’s credibility, one researcher found that 70% of individuals who seek vaccine information online are influenced by what they find on the Internet [1]. Hopefully, patients and parents are able to identify the most reliable resources such as the Centers for Disease Control and Prevention’s (CDC) Vaccine Information Statements (VIS). VISs were developed in response to the National Childhood Vaccine Injury Act (NCVIA) which was passed in an effort to minimize provider liability and respond to public health concerns. The NCVIA requires healthcare providers to provide a VIS to the person receiving the vaccine or his/her guardian. The VIS must be given each time a vaccine is administered and provides information on the benefits and risks of the vaccine as well as other relevant disease information. They are usually provided to the patient as a handout [2].

Some researchers have identified specific issues with the dissemination of vaccine information through these documents. Both Lieu, et al., and St. Amour, et al. have revealed that vaccine documentation handed to patients are rarely read or fully understood by the patient [3,4]. This kind of education is called passive education, because the patients may or may not choose to read the materials. In addition, while the purpose of the VIS is to inform parents and initiate potential questions, limited clinical staff time and resources may hinder the optimal interactions ideal for vaccine education. This demonstrates a pervasive issue with medical education for patients, where the delivery and presentation should be more consumer-friendly in order to effectively impact patient education and increase knowledge [5-7].

Biomedical-related ontologies have had an impact on patient learning and decision-making by utilizing patient-friendly terminology rather than confusing medical jargon. Ontologies are understood to be a consensus-based controlled vocabulary between terms and relationships, while serving as a vehicle of interaction between humans and computers. In lay terms, it is similar to concept maps or graphs but with the capacity to link with other graphs, and evoke reasoning and inferences. Some published examples and ideas include:

- ontology-driven clinical decision support systems for patients in regards to discharge medication [8],
- building medical ontology models for Italian patients [9],
- an ontology-based coaching tool for physicians to prepare dialogue with patients [10],
- and dialogue systems for patient planning [11]

Following in the examples described, we developed an ontology-driven mobile application system to improve patient learning and comprehension of vaccine knowledge [12]. While a proof of concept prototype, the system was limited by the test ontology which comprised of 19 classes and 82 instances, a relatively small knowledge base with which to interact. To further extend this project, as a first step, we aimed to create a comprehensive vaccine ontology with which patients can interact.

The objective of this paper is to formalize the vaccine knowledge for patient education using ontology tools as a solution for vaccine education for the public. We propose modeling vaccine knowledge using OWL2 (Web Ontology Language) [13], which will be encoded with Protégé [14]. We focus on the knowledge in the VISs developed by the CDC. By publishing the Vaccine Information Statement Ontology (VISO) in a domain ontology and offering the benefit of a serialized format that can be processed by a machine and reused [15], we introduce opportunities to develop ontology-driven personal health agents to improve patient learning and comprehension of vaccine knowledge. Overall, the overarching goal is to develop a scalable ontological model that can reliably cover any new and applicable vaccine knowledge for a consumer audience. With a scalable conceptual-level model, opportunities in natural language ontology learning and population would be a future possibility to investigate for automated upkeep and maintenance of the ontology.

This paper will start by briefly discussing the source material, the VISs. Afterwards, the paper will segue way into describing the development of the class-level schema and the instance level of VISO. This will include discussion of the initial common design patterns encountered and basic ontology metrics. The Results section will elaborate the quantitative and qualitative aspects of VISO, that also includes an initial evaluation scoring. The paper will then close with challenges in developing an ontology from the CDC VISs and future direction.

## Materials

The focus of this paper is the ontology representation of the knowledge in the VISs developed by the CDC. Currently there are 25 VISs available from the CDC’s website, and these documents vary between 1 to 2 pages [16]. The VISs describe patient-level information about vaccines ranging from the historical burden of disease to the clinical indications for the vaccine.

The current iteration of the VISO is derived from six CDC VISs:

- Diphtheria, tetanus, and acellular pertussis vaccine (DTaP) [17]
- Rotavirus vaccine [18]
- Hepatitis B vaccine [19]
- *Haemophilus influenzae* type B vaccine (Hib) [20]
- Measles, mumps, and rubella (MMR) [21]
- Pneumococcal conjugate vaccine (PCV13) [22]

All of the vaccines selected are recommended for children on or before the first year of life. Each of the VIS documents are available for download from the CDC’s website in both PDF and RTF format, along with the HTML version.

The VISs are written at a 10th grade reading level [23], and with no images or figures to supplement any passages. Also from a subjective observation, there appears to be some organized consistency of the content within each of the documents, which also helped provide a skeletal structure for the VISO ontology. Some examples of consistent dedicated sections included:

- General vaccine and disease information.
- Possible vaccine reactions and/or side effects.
- Populations for whom the vaccine is indicated.
- General vaccine recommendations and dose information.

## Method

Each of the six VISs acquired from the CDC’s website were examined, and sentences were identified as relevant selections to inform the design of VISO. Ignoring headers and standard text that appeared on VIS documents, the relevant passages were transcribed onto a tracking spreadsheet and coded for simple identification. Any fragment with bullet points was also recorded. Each phrase in the VIS was broken down into a knowledge triple, which is a piece of factual information that is decomposed to a *subject-predicate-object* format that can be visually represented or modeled in an ontology. Separate tracking documents were used for individual VIS documents for management purposes. Later, separate knowledge models for each of the VIS documents were realized, and then collated to obtain a comprehensive model of vaccine information. The proceeding subsections will discuss the high-level conceptualization of the VISO model, detailing the class-level organization and formalization of knowledge, and the last subsection will discuss the encoding of the instances and triples, along with reoccurring design patterns in the VIS domain. The end goal was to develop common classes that can accommodate a large corpus of vaccine information, and have a formalized model to cover an expansive vaccine domain that is relevant for patients.

### Meta-level conceptualization

**Figure 1:**
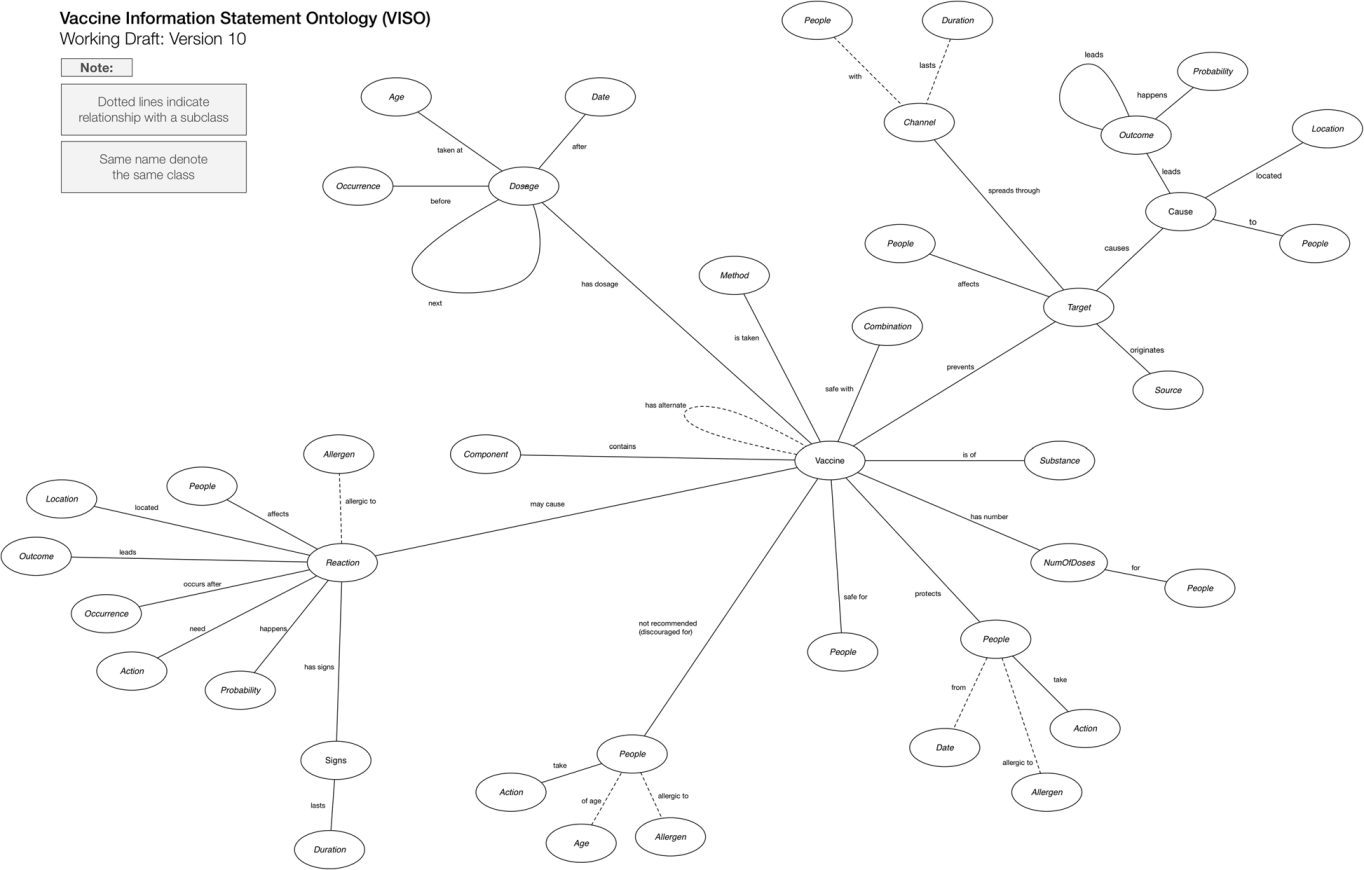
Vaccine Information Statement Ontology (VISO). Parent-level graph of VISO classes.

For a presentation of the properties between the classes see Figure 1. The oval shapes in the diagram depicts the high-level classes in VISO. Between the oval-shaped classes, a line connects two classes to signify a relationship, and a dotted line depicts a relationship between subclasses. Labels are placed beside both the classes and the relationship connection to provide identification. For clarification, some classes in the diagram have the same name, which means they are referring to the same class.

#### Class-level representation

Figure 1 illustrates the meta-level interpretation of the VISO. The model represents a composite of the high abstraction understanding of the six VISs with 23 unique high-level parent classes. Table 1 outlines all of the high-level classes employed for the six VIS document models. The first column lists the formal name of the high-level class, and its corresponding description is listed in the next column. Also, examples of subclasses derived from the class and sample instances are listed in another column, and a count of the number of instances using the class (or using its subclasses) were registered. The most salient classes found in all of the VIS documents were *Vaccine*, *Target*, *Reaction*, *Dosage*, and *People*. The *Vaccine* class designated the vaccine that is the subject of the VIS document. *Target* class referred to what the *Vaccine* class is protecting against, e.g. hepatitis B, rotavirus, etc. The *Reaction* class categorizes any possible side effect or reaction following vaccination. The *Dosage* class is used for any vaccine series and/or dose information from the VIS, and the *People* class organizes various classes of patients who may or may not be recommended to receive the vaccine.

**Table 1:** **Class table**

**Meta-level class**	**Description**	**Example subclasses or** ***Instances (in italics)***	**Instances**
Vaccine	A class description to categorize vaccines documented by the CDC’s VIS documentation	AlternateVaccine	9
Target	Provides class specification for virus, bacteria, diseases, etc. that are prevented by Vaccine Disease	Virus, SeriousDisease, Bacteria	12
People	Categorizes various types of patients or groups of individuals impacted by vaccines, diseases, reactions, and other health conditions	PeopleWithCondition, Infants, PeopleWithIllness, PeopleWithModerateIllness, PeopleOfAge, Children, Adults	93
Source	Used to describe an origin of a vaccine’s target (bacteria, disease). Can be reused in relations with other classes.	*bacteria, Hepatitis B virus*	5
Channel	Class of medium of travel for vaccine targets.	PeopleChannel, ObjectChannel, PeopleWithConditionChannel, DermalChannel, HumanActivityChannel, FluidChannel s	19
Cause	For a description of a condition as a result of a vaccine target. E.g. infection, coughing spells.	*ear infection, long-term illness, coughing spells, pneumonia*	39
Location	Type to categorize location, specifically area of the body, affected by a heath condition or reaction	FacialLocation, ThroatLocation, ArmLocation	14
Probability	Classification for types of probabilities enumerated in VIS documents	QualitativeProbability, QuantitativeProbability, ProbabilityInCases	38
Outcome	Types of effects resulting from causation, reactions, or chain of outcomes.	RareOutcome, AdultOutcome, ChildOutcome, FatalOutcome	38
Duration	Used for various types of descriptions for qualitative length of time for effects of health conditions or signs of conditions.	DurationInMinutes, DurationInWeeks	5
Substance	Classification of kinds of substance for vaccines and possibly other class groupings.	LiquidSubstance, GaseousSubstance, SolidSubstance	1
Combination	For various artifacts that interact with vaccines.	SafeCombination, DangerousCombination	1
Method	Groups and classifies inoculation methods for vaccines.	InhaleMethod, InjectionMethod, OralMethod	1
NumberOfDoses	Enumerates the maximum number of doses for vaccine.	OptionalNumberOfDoses	6
Dosage	Designates the types of dose or the dose interval. E.g. 1st Dose, 3rd Dose.	OptionalDose, DoseIntervals	23
Component	Categorizes types of elements of a vaccine.	ViralComponent, NoninfectiousViralComponent	3
Age	Enumerates the type of quantitative classification of age ? years, months, weeks, etc.	AgeInMonths, AgeInYears	25
Date	Enumerates the types of quantitative classification of date ? days, months, weeks, etc.	DateInYears, DateInMonths, DateInDays	4
Occurrence	Classification of types of events	VaccinatedOccurence, TimedOccurence	24
Action	Types of patient recourse in response to reactions or actions required vaccine patients before inoculation.	InactiveAction, ActiveAction, EmergencyAction	8
Allergen	For classes of substances leading to an allergic reaction.	VaccineAllergen, VaccineComponentAllergen	7
Reaction	Used to categorizes types of reactions that may result from vaccination.	MildReaction, TemporaryReaction, SeriousReaction, ModerateReaction, AdultReaction	71
Sign	Class of indicators for vaccine reaction effects.	*fast heartbeat, crying, stomach pain, hives*	14
**Total instances of classes**			**460**

#### Subclass-level representation

The VISO model’s high-level classes utilizes extensive subclassing to cover specific abstraction of entities. One apparent use was for categorization purposes to organize the knowledge for better identification, such as the case for the *People* class. For example, the VIS documents often refer to a specific population that may react differently to vaccines, or may need a different vaccine type due to age or certain medical conditions, justifying the need to create subclasses to refer to specific groups of people.

Another motivation for subclasses is to facilitate descriptions from the text that are in the form of adjectives or prepositions. If the documentation refers to the number of optional doses of a vaccine, a subclass called *OptionalNumberOfDoses*, which is a child of *NumberOfDoses*, was defined. Attention was given to classes that have “universal” subclasses that may not have been observed from the VIS documentation. Some examples are *Organ* class (“*Heart*”, “*Liver*”, “*Lungs*”) or *Substance* class (“*GaseousSubstance*”, “*LiquidSubstance*”, “*SolidSubstance*”).

#### Value sets and partitions

To address quantitative, descriptive, or ranks in classes, value set representations, as described in a W3C working draft [24], was utilized for the VISO meta-ontology. More specifically, we use subclasses to represent permissible values in a value set. For example, the VIS documentation alluded to a three-point scale, especially when describing severity of conditions - *mild, moderate, severe* (or *serious*). We created a *Reaction* class that has the subclasses *MildReaction*, *ModerateReaction*, and *SeriousReaction* to describe the degree of vaccine effect severity. Other classes that employ value sets include the *People* class and the *Target* class. For any other units of measure revealed in the VIS documents, specific classes yielded subclasses that handled units of measure, such as, *AgeInYears* and *AgeInMonths* for the *Age* class or *DateInMonths* and *DateInDays* for the *Date* class.

#### Properties

Knowledge triples evoked by the VIS documents suggested common relationships or properties between classes across the VIS corpus. In all of the 6 documents, it was common to describe a vaccine preventing a bacterial infection or virus, or a vaccine can potentially cause a rare reaction following administration. Many of these properties between the classes were identified and normalized to a standard representation. One example in the DTaP VIS, the evoked triple - “*Another vaccine, called Td, protects against tetanus and diphtheria...*” - utilized the predicate “*protect against*”, which essentially means “*Td prevents tetanus and diphtheria*”. In result, “*Td protects against tetanus*” was rendered as “*Td prevents tetanus*”, where “*prevents*” is the standard property, or controlled term, to describe that specific relationship.

Table 2 identifies all of the object properties utilized in VISO. The first column list the domain classes, with the properties and range classes in the subsequent columns. Overall, 33 types of object properties exist in the current iteration of VISO.

**Table 2:** **Object properties**

**Domain**	**Properties**	**Range**	**Triples**
Target, Cause, Reaction	affects	People, Location	11
Dosage	after	Date	2
PeopleWithAllergicReaction, AllergicReaction	allergic to	Allergen	7
Dosage	before	Occurrence	1
PeopleBornFrom	born from	Date	1
Target	causes	Cause	40
Vaccine	contains	Component	3
Vaccine	discouraged for	People	19
NumberOfDoses	for	People	5
Cause, Outcome, Reaction	happens	Probability	42
Vaccine	has alternate	Alternate Vaccine	4
Vaccine	has dosage	Dosage	26
Vaccine	has number of	NumberOfDoses	6
Reaction	has signs	Sign	14
Vaccine	is a substance of	Substance	1
Vaccine	is safe for	People	6
Vaccine	is safe with	Combination	1
Vaccine	is taken	Method	1
Target, Reaction, Sign, ObjectChannel	lasts	Duration	5
Target, Cause, Outcome, Reaction	leads	Outcome	31
Cause, Reaction	located	Location	15
Vaccine	may cause	Reaction	54
Reaction	need	Action	3
Reaction, Sign	occurs after	Date, Occurrence	24
PeopleOfAge	of age	Age	3
Target	originates	Source	5
Vaccine	prevents	Target	11
Vaccine	protects	People	23
Target	spreads through	Channel	19
People	take	Action	5
Dosage	taken at	Age	22
Cause	to	People	2
HumanActivityChannel	with	People	2
**Total number of triples**			**414**

Table 3 list data properties. Similar in format to Table 2, two types of data properties are used in VISO with the domain listed as Protégé’s superclass *Thing* and a string literal for its range. These data properties are meant to be global to all of the classes. These two properties serve as utility properties to accommodate information that either provide an alternative name (“*also known as*”) or information that describe or define an object of the class (“*is described as*”).

**Table 3:** **Data properties**

**Domain**	**Properties**	**Range**	**Triples**
Thing	also known as	string data	7
Thing	is described as	string data	8
**Total number of triples**			**15**

### Instances and triples

Afterwards, the meta-level ontology development led into the encoding of instances from the collected VISs. Referring to Table 1, the *People* class accounted for most of instances, with 93 instances of the *People* classes. Since the initial set of VIS documents was small, some of the classes had one instance stemming from its class. There is a possibility that the remaining VIS documents may add more instances to these classes.

Earlier, we indicated that across the VIS documents there exist some consistency that influence the representation of knowledge in the VISO. This was also reflected in how the instances are conceptualized. The following subsections list some noticeable design patterns observed in the development of VISO - *dosage pattern* for vaccine dose representation, *target pattern* for representing the object of vaccine prevention, and the *reaction pattern* for possible side effects of the vaccine.

#### Dosage pattern

**Figure 2:**
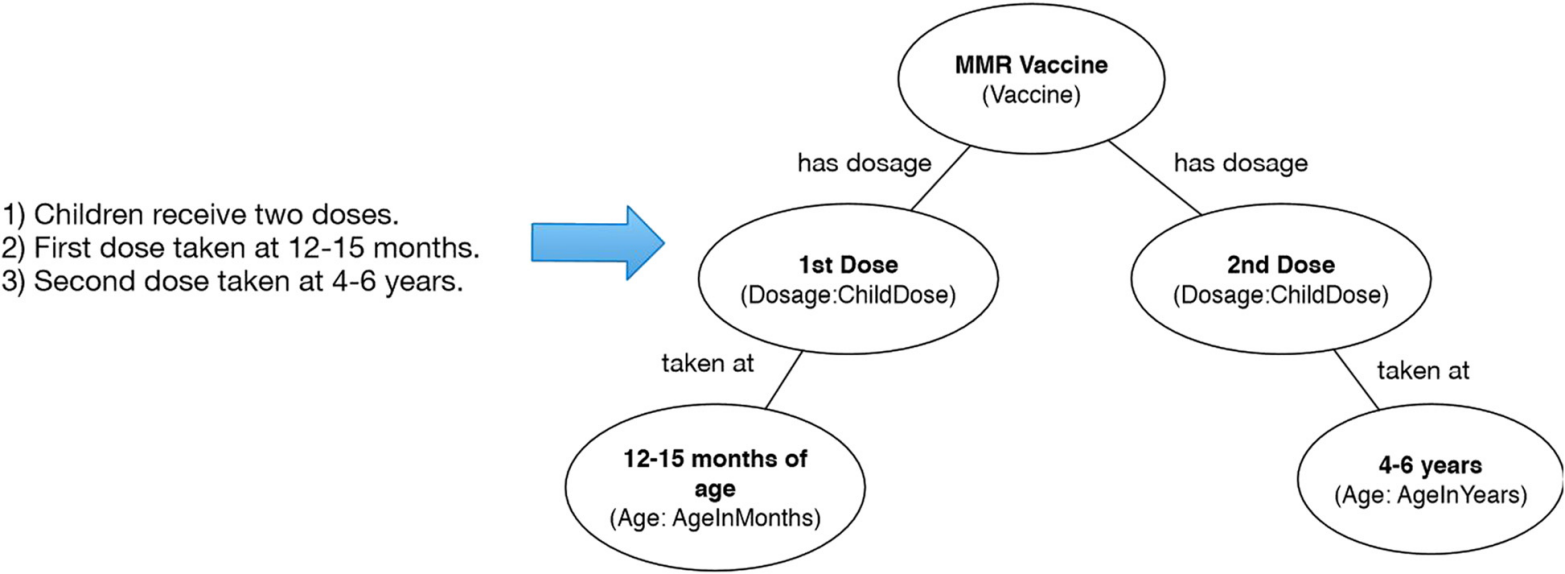
Dosage modeling example. Example of Dosage instance representation with an excerpt from the MMR VIS.

Every vaccine documentation outlines the number of doses, and when and who should receive the vaccine. The three knowledge triples provided in the representation in Figure 2, where statement 1 is represented as a *ChildDose* subclass, and their corresponding dose order (statements 2 and 3) are depicted in subclasses of *Age* for the the time of the vaccination. Most of the dose information found in the VIS documents are modeled similarly in VISO.

#### Target pattern

**Figure 3:**
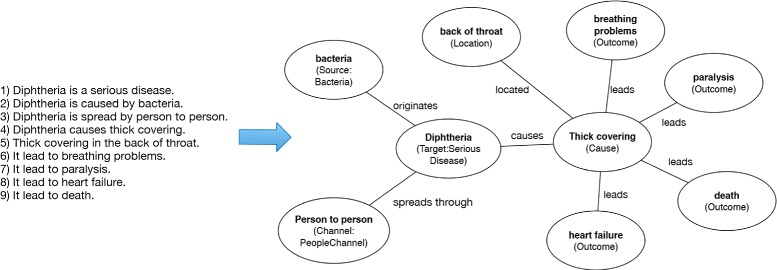
Target modeling example. Example of Target instance representation with an excerpt from the DTaP VIS.

Pertinent information on what a vaccine protects against is found in the six vaccine statements. *Target* instances comprise of measles, tetanus, pneumococcal disease, rotavirus, pertussis, etc. Figure 3 illustrates a typical pattern for *Target* instances. In the figure, the 9 statements listed are mapped in accordance to the VISO model. Statement 1 reveals the type of *Target* (“*Serious Disease*”) that diphtheria is.

Statement 2 is abstracted with a *Source* subclass of *Bacteria*, with a relationship of “*originates*” with the *Target* instance. Likewise, statement 3 is shown as *PeopleChannel* subclass of *Channel*. Statements 5 through 9 are facilitated by the *Cause* class with relationships to instances of *Location* and *Outcome* classes. This is an example is indicative of how *Target* class instances and their commonly related relationships are modeled in VISO.

#### Reaction pattern

**Figure 4:**
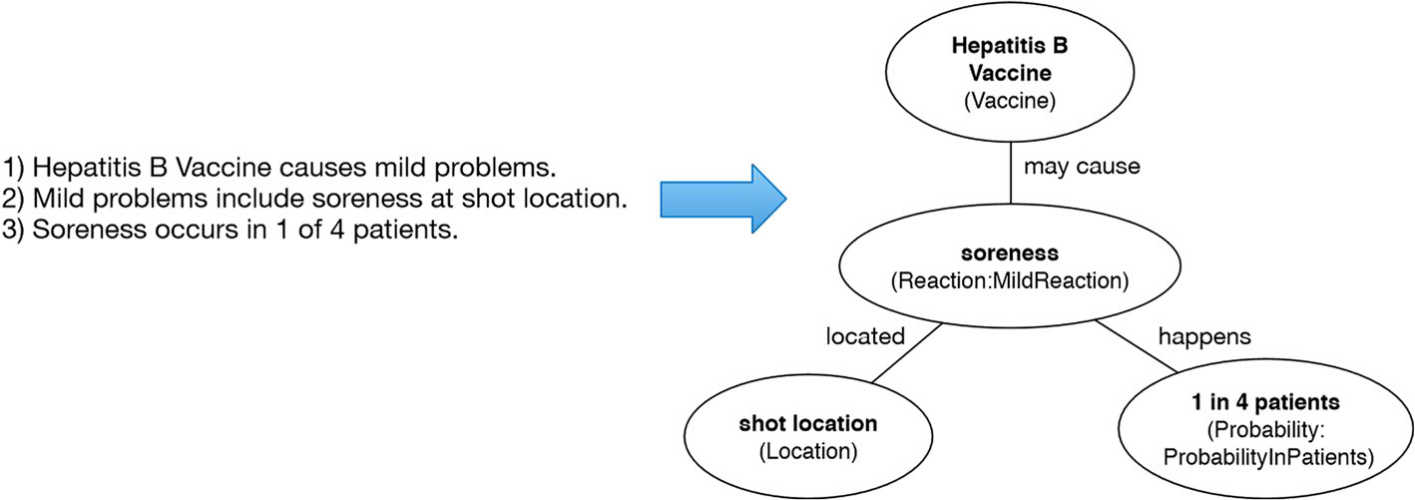
Reaction modeling example. Example of Reaction instance representation with an excerpt from the Hepatitis B VIS.

Reaction patterns modeled possible reactions from vaccinations described in each of the VIS documents. Figure 4 shows a model of three sample statements. Statement 1 shown as an instance of *Vaccine* may cause *MildReaction* triple. Statement 2 alludes to the left branch with an instance of *MildReaction* located at *Location*, and statement 3 represented in the right branch of a *Probability* subclass of *ProbabilityInPatients*.

The patterns described in this section are the most evident types in the mapping of the information, but additional vaccine information design patterns also exist. It is also understood that more patterns may emerge with the inclusion of the remaining VIS documents.

### Quality evaluation

To evaluate the overall quality of VISO, the ontology was scored using the ontology metrics suite proposed by Burton-Jones, et al. [25]. Inspired by semiotics concepts, this simple, yet extensive ontology scoring metric evaluates an ontology based on four criteria - *semantic*, *syntactic*, *social*, and *pragmatic* levels. The semantic criteria evaluates the term meanings and word sense of each of the terms. The syntactic criteria assesses syntax of the ontology, while social criteria examines how other ontologist use the ontology through links. The pragmatic criteria evaluates the practicality of the ontology, relating to its usage and its construction. Also, the evaluation metric is flexible to allow individual tailoring if any of the criteria does not apply, as demonstrated in reference [25]. The resulting final score is a value between 0 and 1.

Because of the initial design of the ontology, the scoring metric needed to be tailored to provide a progress indicator of its development. For example, social criteria which is dependent on other ontologies linking to VISO was not possible to determine, therefore, it was excluded from the complete calculation score.

However, the attainment of the other scores were straightforward. The semantic quality score (*E* in equation (1)) was attained by a normalized summation of *“Interperatability”* (*w*_1_ as terms with a word sense in WordNet/ *TC* as total terms used in the ontology), *“Consistency”* (misused terms as *i*/total classes, properties, and instances as *C*), and *“Clarity”* (average number of word senses for each term, *w*_2_/total classes, properties, and instances, *C*). For *w*_1_ (*“Interperatability”*) and *w*_2_ (*“Clarity”*), a simple Java GUI-based application utilizing the ws4j library for WordNet [26] and the OWL API libray [27] was developed to automate and retrieve the values to calculate semantic scores. Equation (1) outlines the semantic quality calculation.

(1) $$\begin{array}{@{}rcl@{}} E = \left(\frac{1}{3}\ast\frac{w_{1}}{TC}\right)+ \left(\frac{1}{3} \ast \frac{i}{C} \right) + \left(\frac{1}{3} \ast \frac{w_{2}}{C} \right) \end{array} $$

The pragmatic score, *P*, was determined by the normalized summation of *“Comprehensiveness”* (*C*/500 as total number of classes and properties over 500, the maximum number for classes and properties for the calculation [25]), and *“Accuracy”* (false statements as *f*/total number of statements from the ontology as *TS*). *“Relevance”* scoring (application of statements/total number of statements) involves ontology usage in an application, which was omitted. For *“Accuracy”*, vaccine subject matter experts were given a list of statements from the ontology and were asked to label whether the statement was true or false and to provide any corrections. While the terminology and the knowledge source was primarily from the VIS, our vaccine experts also interact directly with patients and their concerns relating to vaccines which helped to focus on the patient-centric goal of the VIS. Below, equation (2) describes the calculation of *P*.

(2) $$ P = \left(\frac{1}{2} \ast \frac{C}{500} \right) + \left(\frac{1}{2} \ast \frac{f}{TS} \right)   $$

For syntactic quality, *S*, the Protégé editor provided some of the values for the scores. Both the *“Lawfulness”*, number of syntactic violations over total number of statements in the ontology (*l*/*TS*) and *“Richness”*, number of syntactic elements utlized over the total syntactic elements (*s*/*SF*) accounted for the normalized sum of syntactic quality. Equation (3) shows the syntactic quality calculation.

(3) $$ S = \left(\frac{1}{2} \ast \frac{l}{TS} \right) + \left(\frac{1}{2} \ast \frac{s}{SF} \right)   $$

While the metric evaluation can be tailored specific to the ontology, aspects like the social criteria and *“Relevance”* were excluded. The modified final score is represented in equation (4), where *Q* is the overall quality score.

(4) $$ Q = \left(\frac{1}{3} \ast E \right) + \left(\frac{1}{3} \ast P \right) + \left(\frac{1}{3} \ast S \right)   $$

## Results and discussion

### Results

The VISO was serialized in OWL2 with Protégé. The metrics data collected from Protégé is shown in Table 4. The first column displays the item and the second column displays the value associated. The current VISO model produced a class count of 132, including subclasses; 33 object properties; 2 data properties; and 419 unique individual instances. In addition, 460 instances derived from concepts were created, along with a total of 429 knowledge triples.

**Table 4:** **VISO metrics data**

Class count	132
Object property count	33
Data property count	2
Unique individual count	419
Instances asserted from classes	460
Total knowledge triples	429

As stated earlier, part of the process in engineering the abstraction of the VIS involved obtaining passages to be mapped into the model. Table 5 displays the number of passages parsed for evaluation under the “Total Passages” column. Also, the third column recorded the number of instance triples produced. For all six of the VIS document sources, a total of 244 sentences and passages were used, and 427 instance triples were extrapolated from them, and later merged and coded with Protégé to VISO.

**Table 5:** **Extraction Results**

**CDC VIS**	**Total passages**	**Instances of triples in VISO**
Hepatitis B	45	76
Rotavirus	49	64
Hib	41	43
PCV13	32	50
DTaP	51	112
MMR	26	82
**Total**	**244**	**427**

Table 2 indicate the number of knowledge triples present in the current VISO version. Most of the triples were mainly of the “*Vaccine may cause Reaction*” type, with 54 instances of that triple. Total number of instances of triples in VISO numbered at 429 (derived from Tables 2 and 3), that includes the 15 instances of triples denoted by data property relationships from the table in Table 3.

Both the HermiT (version 1.3.8) and the FaCT+ reasoners with the Protégé editor did not reveal any discrepancies with this version of the model.

The final quality score for evaluation, *Q*, based on the tailored equation (4) calculated to 0.54. Observing the other components of the quality score, the semantic quality, *E*, amounted to 0.59. Pragmatic quality and syntactic quality equated to 0.75 (*P*) and 0.27 (*S*), respectively.

### Result analysis

While the construction of VISO is in the early stages and covering 6 VISs, the quality score appears to reflect the early developmental state of the ontology. However, looking at the decomposition of the score can reveal some insight to VISO and possibly inform the future direction of the VISO development.

Observing specifics of the semantic quality, the *“Clarity”* component score (0.89) reveals less ambiguity among the terms. The *“Interpertability”* value (0.74) also indicates the use of meaningful terms in the ontology, and the inconsistency of the terms was low (0.04).

With pragmatic quality (0.75) of VISO, we ignored the *“Relevance*” quality due to unavailability for deployment in an application environment. *“Comprehensiveness”* quality (1.2) was exceedingly high due to being a large ontology with many classes and properties. Based on reports from two expert reviewers, VISO’s inaccuracy was relatively low at 0.30.

Syntactic quality revealed no violations with syntax, however, the *“Richness”* quality computed at 0.54, reflecting that VISO was utilizing a little over half of the syntactic features available.

Based on the results, the VISO’s strength lies in its pragmatic quality which indicates its overall usability based on the two of the three components described earlier - *“Accuracy”* and *“Comprehensiveness”*. The excluded component, *“Relevance”*, could provide a more holistic score in the future. Syntactic outcome was the weakest of the three aspects of VISO’s quality. This is partially due to the minimal usage of ontology features available. Focus on this aspect will warrant attention in future development of VISO.

Relating to *Consistency*, some terms, according to reviewers, were improperly used or required nuanced descriptions. One example is the term “*Dosage*” which is technically used to describe measurement of a vaccine and not a synonym for “dose”. Another example are property labels like “*cause*”. Some instances in the ontology should have been labeled *“may cause”* to imply a possible causation, rather than an expected outcome. Proper and precise term usage will be another focus that the next version of VISO will rectify.

### Discussion

Modeling the CDC’s VISs posed several challenges. One challenge was determining relevant pieces in the corpus that could be used for knowledge extraction. Most of the documents had statements that were either repetitive, or had literary flourishes that were deemed unnecessary. Also, the documents may have a paragraph or a sentence that summarizes preceding information with granularity. In most cases, these were viewed as repetitive, yet may serve a future purpose if the ontology were to be used to construct dialogue with patients. Additionally, the documentation comprised of some knowledge that were historical statements. An example from the rotavirus VIS:

*Because children are protected by the vaccine, hospitalizations, and emergency visits for rotavirus have dropped dramatically.* [18]

It is debatable whether the historical information may be useful to patients, or if summarized statements, which are naturally repetitious in these documents, could be integrated into the VISO model. In this initial version of VISO the repetitive and historical texts were not mapped but may be considered in future versions of the ontology.

Another challenge were gaps in the knowledge where the information was incomplete, brief, or needed medical understanding beyond the lay person. Some of the language in the documents may not be readily evident to a parent or patient, and would require a medical professional to provide interpretation. For example, if a certain vaccine should not be given to a child who is “moderately ill”, how does the reader of the VIS determine the exact signs of a “moderately ill” child as opposed to a child who may be “mildly ill”? If the documents refer to a sign as “physical weakness”, how does the patient or the reader determine features or indicators of “physical weakness”? Issues like these limited the scope of the VISO knowledge-base. In practice, the healthcare provider, rather than the parent, will determine if the child is too ill to receive the vaccine. It is the medical professional’s responsibility to provide judgment and guidance to the patient. Similarly, there were issues with limited number of VIS documents to develop the VISO models and to create instances. Lack of additional information resulted in some of the classes not having any subclasses to suggest.

Expressing the knowledge contained within the VIS documents posed some challenges as well. While the meta-level definition was designed with subclasses to handle descriptive instances, there are often passages with complex nouns and adjectives where each word carried important meaning for the instance. Examples such as “painful tightening of muscles” or “difficult for infants to breathe” posed a predicament of whether these instances should be decomposed to additional classes and relationships; use a subclass or create a new subclass; apply polymorphism; or keep as is as an instance. In most cases, they were realized as a single instance, until the meta-level model is further developed to map difficult passages. Moreover, given the historical nature of the VISs and considering that several VISs were originally developed more than 30 years ago in response to NCVIA, it is assumed that subsequent versions contain much of the original language. This likely contributes to the variability in the semantic language of the ontology.

## Conclusion and future direction

We introduce the Vaccine Information Statement Ontology, which could positively influence the development of intelligent ontology-drive applications and mitigate the knowledge gap that often exists in patients seeking accurate and reliable information but encountering complex or inaccurate sources. Possible future goals in continuing the development of the VISO include:

- *Expand the Vaccine Information Statement Domain.* This version of the VISO models 6 VIS documents as an initial iteration to examine the ontology. The next few iterations of VISO will include more domain knowledge from the remaining 19 VIS documents, available from the CDC website. There is also an awareness that additional knowledge can be modeled from outside the CDC’s VIS documents. It is also assumed that the meta-level design will mature as we realize alternative interpretation of vaccine information, or discover abstractions that could integrate some of the ignored passages and phrase, like negation statements or summary passages. With an expanded version and throughout the development cycle, we plan to evaluate the ontology using semiotic-based metric suite, and also adapt the suite to also include aspects previously excluded, like *social* quality or *Relevance*.
- *Link VISO with existing relevant ontologies to expand the knowledge domain.* Some third party ontologies that could be aligned with the VISO may include the Vaccine Ontology (VO) [28], Ontology of Vaccine Adverse Effect (OVAE) [29] or Medical Dictionary for Regulatory Activities (MedDRA) [30] to address reaction or conditions in the VISO. This approach would comprise of code-linking particular classes with matching classes in the VISO model, which would lead into providing a comprehensive and expanded knowledge-base for patient learning. However, because the knowledge-base is intended for patient use, it will be essential to determine the appropriate since the aforementioned third party ontologies utilize professional vocabulary which may not be understood by patients.
- *Integrating patient-level synonymous terms and multi-language equivalents into the lexicon.* A multi-lingual VISO would presumably expand to reach potential patients who may be excluded because of language or socio-economic barriers. There is also an interest in using the Open Access, Collaborative Consumer Health Vocabulary Initiative [31] as a resource to integrate consumer-level terms or synonyms.
- *Applying natural language processing.* We intend to explore the possibility of applying natural language processing (NLP) for both ontology learning and knowledge retrieval. We will implement NLP methods to facilitate automatic knowledge extraction to expand the VISO. This will also provide intrinsic value for applications using natural language processing and dialogue systems.
- *Intelligent mobile agents.* Ontology-driven applications could introduce the potential for intelligent agents for learning. Realistic and engaging agents are proven to be more effective for increasing involvement and learning [32-35], persuasion [36], and trustworthiness [37,38]. It provides a cost-effective way to address patient’s concerns and answer their questions about vaccination, which otherwise requires healthcare professionals to address in person. This will presumably improve the efficiency of healthcare delivery workflows and reduce the cost. They also provide flexibilities for vaccine education. Patients or parents can spend as much time as they need with intelligent mobile devices, and interact with intelligent devices anytime they have access to a computer or tablet. Last but not least, personalized agents can be automatically built according to users’ preference to improve the usability and acceptability of the system.
  Examples like VAMATA, which is an ontology-driven mobile application with a speech interface designed for combat medical settings, reveal applicable synergy between natural language processing and ontology in mobile applications [39].

We have previously developed a proof-of-concept ontology-driven mobile application with a natural language interface to query a VISO knowledge-base [12]. The ongoing evolution of VISO will assist in the continuing development of that project.
